# P22 Youth as agents of change in raising AMR awareness in the community in Dodoma region

**DOI:** 10.1093/jacamr/dlac004.021

**Published:** 2022-02-16

**Authors:** Erick Venant, Baritazar K. Stanley, Michael J. Mosha, DorineGrace J. Mushi, Israel Mwingira, Roger Harrison, Karin Wiedenmayer, Eva Ombaka

**Affiliations:** RBA Initiative, Dodoma, Tanzania; RBA Initiative, Dodoma, Tanzania; St John’s University of Tanzania, Dodoma, Tanzania; RBA Initiative, Dodoma, Tanzania; St John’s University of Tanzania, Dodoma, Tanzania; University of Manchester, Manchester, UK; Swiss Tropical and Public Health Institute, Basel, Switzerland; University of Basel, Basel, Switzerland; St John’s University of Tanzania, Dodoma, Tanzania

## Abstract

**Introduction:**

One of the strategic objectives of the AMR national action plan is to improve public awareness and understanding of AMR. Changing people's behaviour is not easy and the older a person is, the more they are set in their ways. Schoolchildren are in their formative years, which is the right time to impart knowledge and best practices that will guide their behaviour in life. Importantly, students have links with families and communities and are future leaders. Roll Back Antimicrobial resistance Initiative (RBA Initiative) has been pioneering the use of youth as agents of change to increase AMR awareness, promote positive behavioural change and thus reduce treatment failure.

**Objectives:**

The main objective was to equip young people (schoolchildren) with knowledge and skills to understand antimicrobial use and resistance and ability to pass the knowledge to their families, other students and community at large.

**Methods:**

Through AMR clubs, RBA Initiative used different methodologies to engage and educate schoolchildren on AMR. These included classroom teaching, arts and crafts like songs, skits, drama, traditional dances and storytelling and fun videos. Further motivation for active participation was encouraged through competition. The content of the training included topics such as behaviour that fuels AMR, hand hygiene and sanitation, the impact of fake drugs and the One Health approach.

**Results:**

In 2020, the project build the capacity of 160 students. Subsequently, these trained students have reached over 1000 fellow secondary school students, over 3000 primary school pupils and over 800 community members including family members with key AMR messages.

**Conclusions:**

If engaged and empowered, young people are able to increase community knowledge and awareness regarding AMR as agents of change, contributing to the national action plan on AMR.

